# Three-Dimensional Anisotropic Magnetoresistance in the Dirac Node-Line Material ZrSiSe

**DOI:** 10.1038/s41598-018-27148-z

**Published:** 2018-06-19

**Authors:** Haiyang Pan, Bingbing Tong, Jihai Yu, Jue Wang, Dongzhi Fu, Shuai Zhang, Bin Wu, Xiangang Wan, Chi Zhang, Xuefeng Wang, Fengqi Song

**Affiliations:** 1 National Laboratory of Solid State Microstructures, School of Physics, Nanjing University, Nanjing, 210093 China; 2 International Center for Quantum Materials, Collaborative Innovation Center of Quantum Matter, Peking University, Beijing, 100871 China; 3 School of Electronic Science and Engineering, Nanjing University, Nanjing, 210093 China; 4 Collaborative Innovation Center of Advanced Microstructures, Nanjing University, Nanjing, 210093 China

## Abstract

The family of materials defined as ZrSi*X* (*X* = S, Se, Te) has been established as Dirac node-line semimetals, and subsequent study is urgent to exploit the promising applications of unusual magnetoresistance (MR) properties. Herein, we systematically investigated the anisotropic MR in the newly-discovered Dirac node-line material ZrSiSe. By applying a magnetic field of 3 T by a vector field, three-dimensional (3D) MR shows the strong anisotropy. The MR ratio of maximum and minimum directions reaches 7 at 3 T and keeps increasing at the higher magnetic field. The anisotropic MR forms a butterfly-shaped curve, indicating the quasi-2D electronic structures. This is further confirmed by the angular dependent Shubnikov-de Haas oscillations. The first-principles calculations establish the quasi-2D tubular-shaped Fermi surface near the *X* point in the Brillouin zone. Our finding sheds light on the 3D mapping of MR and the potential applications in magnetic sensors based on ZrSiSe.

## Introduction

Recently, Dirac semimetals have attracted extensive attention due to their unusual linear band crossing at fourfold degenerate Dirac points between the valence band and the conduction band in the Brillouin zone (BZ)^1–7^. These unique electronic band structures lead to many intriguing transport phenomena. For instance, the large non-saturating magnetoresistance (MR)^8,9^, negative MR^10,11^ and Aharonov-Bohm oscillations^12^ have been observed in Dirac semimetal materials. Moreover, the extended lines or closed loops of the crossing Dirac points in the BZ form the interesting Dirac node-line materials (DNLMs)^13–21^. The Dirac node-line electronic state contributes to a high carrier density of ~10^21^ cm^−3 ^^22,23^, significantly larger than those of graphene and other Dirac semimetals^1–3,6,24^. PbTaSe_2_^25^, PtSn_4_^26^ and the *WHM* (*W* = Zr/Hf/La, *H* = Si/Ge/Sn/Sb, *M* = O/S/Se/Te)^27^ compounds were theoretically predicted and experimentally proved to be the typical DNLMs^28–34^. Particular attention has been paid to materials in the *WHM* family due to the fact that they exhibit the space group of iron-based superconductors and their monolayers are suggested to be possible two-dimensional (2D) topological insulators^27^. The diamond-shaped Fermi surface hosting line nodes and the symmetry-protected bands indicate the formation of the node-line semimetal (NLSM) phase in ZrSi*X* (*X* = S, Se and Te) family^28,29,31,34–37^. The nearly electron-hole-compensated carriers result in the high MR ratio^38^ as well as the large anisotropic MR (AMR)^39–41^. The AMR is important to realize magnetic sensors, which offers the possibility to explore the important technological applications of NLSMs^42^.

Up to now, the AMR has been observed in many topological materials, such as WTe_2_^43,44^, NbP^45^ and LaBi^46^. The MR of fixed magnetic field value in ZrSi*X* (*X* = S, Se, Te) shows the clear butterfly-shaped anisotropy when changing the direction of magnetic field^22,38,39,41^. However, all these AMR measurements were carried out in a 2D plane, limiting insights into the complete AMR effect in these Dirac semimetals. It remains yet unexplored that 3D mapping of MR in NLSMs, which is of vital importance for potential magnetic sensor device applications.

In this work, we systematically investigated the AMR in the newly-discovered Dirac NLSM ZrSiSe. The 3D mapping of the MR exhibits the strong anisotropy. The minimum and maximum of MR is observed when the magnetic field sweeps along [100] and [011] family directions, respectively. The AMR curves in *bc*- and *ac*-planes show the butterfly shape, indicating the quasi-2D electronic structure. The 2-fold and 4-fold symmetries of the butterfly-shaped MR are robust at the high magnetic field. The angular dependent Shubnikov-de Haas (SdH) oscillations further depict a quasi-2D Fermi pocket with the frequency of 210 T. This establishes the tubular-shaped Fermi surface near the *X* point in the BZ. The AMR behavior is associated with the quasi-2D Fermi surface structure. Our work provides a route to realize magnetic sensor devices based on the anisotropic Fermi surface of NLSMs.

## Results

**Figure 1:**
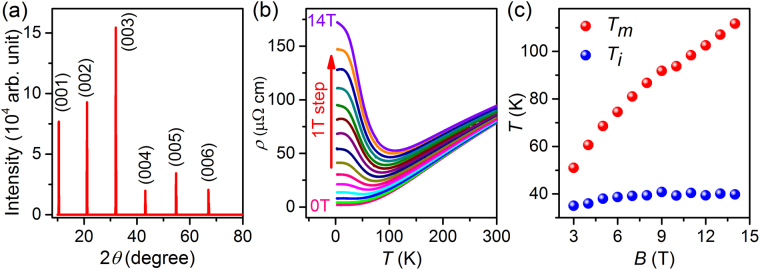
Crystal structure and temperature-dependent resistivity of ZrSiSe bulk crystals. (**a**) X-ray diffraction patterns of the typical ZrSiSe. (**b**) Temperature-dependent electrical resistivity at 0 T and magnetic field up to 14 T. The magnetic field is applied along [001] direction. (**c**) Curves of *T*_*m*_ (where the resistivity reduces to minimum) and *T*_*i*_ (where the resistivity reaches to a saturation plateau) versus the magnetic field.

Figure 1(a) shows the X-ray diffraction pattern of the exposed plate-like surface of a ZrSiSe single crystal. The presence of sharp (00 *l*) peaks can be attributed to the (001) plane (*c*-plane) of the plate-like surface and the high crystallinity of ZrSiSe crystals. As shown in Fig. 1(b), the temperature-dependent resistivity at zero magnetic field exhibits the metallic behavior and almost reaches saturation below 50 K. When a magnetic field is applied along *c*-axis, the temperature-dependent resistivity increases at low temperature and undergoes a drastic enhancement (*B* > 3 T), which results in a minimum resistivity. This behavior is very similar to the metal-insulator transition, which is observed in many topological semimetals with the ultrahigh mobility and the large MR, such as NbP^45^, WTe_2_^9,44,47^ and LaBi^46^. The resistivity saturation plateau also appears at the low temperature with the different external magnetic fields. To analyze the resistivity transition, we plot the curves of *T*_*m*_ (where the resistivity reduces to minimum) and *T*_*i*_ (where the resistivity reaches to a saturation plateau) versus magnetic field in Fig. 1(c). It is evident that *T*_*m*_ keeps monotonous increase with increasing magnetic field while *T*_*i*_ is almost unchanged.

**Figure 2:**
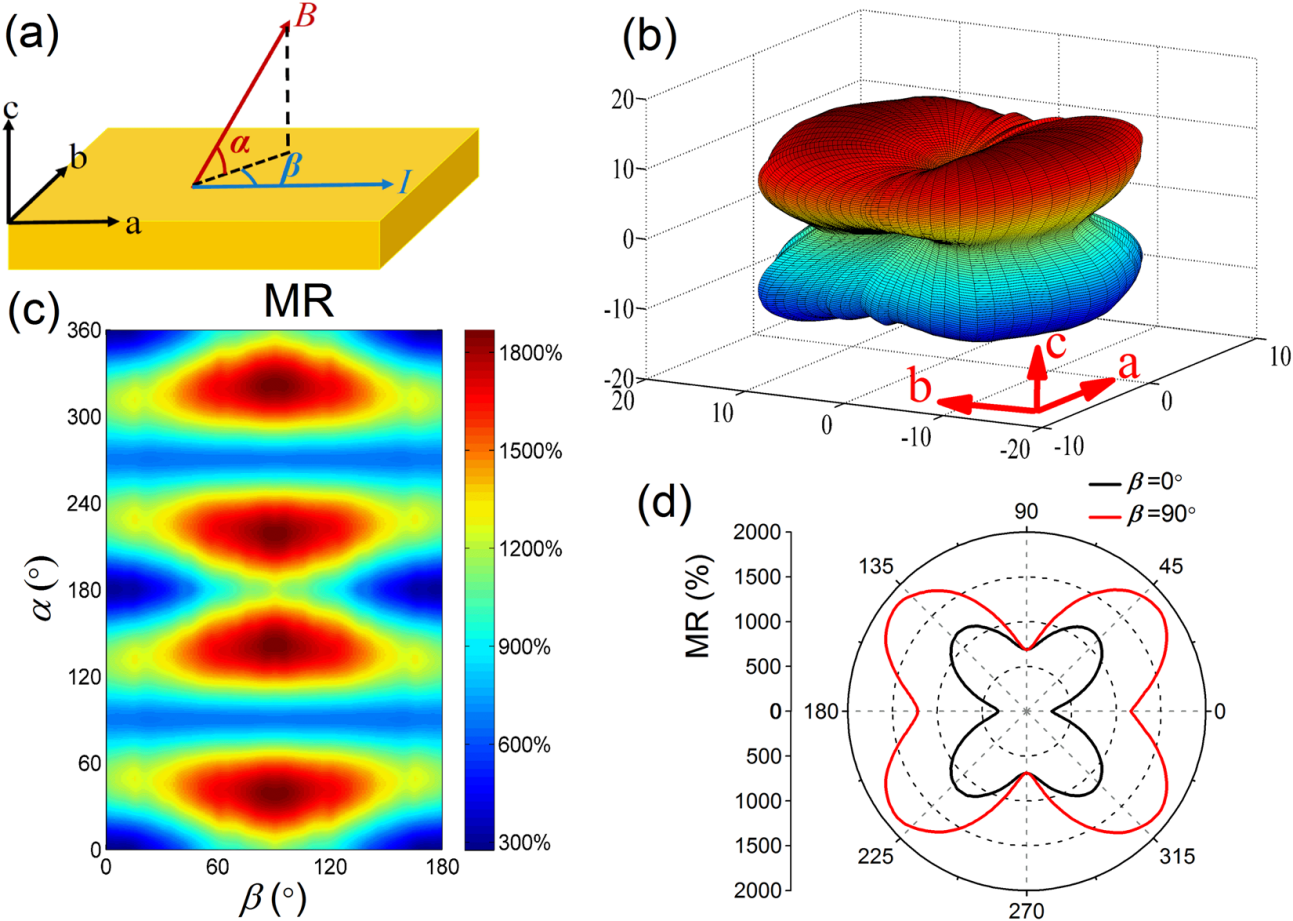
3D-space MR measurements of ZrSiSe at 1.7 K. (**a**) Schematic diagram of 3D-space MR measurements. The current is applied along *a*-axis. *α* is the angle between the magnetic field and *c*- plane. *β* is the angle between the current and the projection direction of the magnetic field. (**b**) The 3D-space MR mapping. (**c**) Corresponding color plot of the 3D-space MR. (**d**) The polar plot of angular dependent MR of 3 T at *β* = 0° (*ac*-plane) and *β* = 90° (*bc*-plane), respectively.

As previous reported, the MR of fixed magnetic field values in ZrSi*X* (*X* = S, Se, Te) shows the clear butterfly-shaped anisotropy when changing the direction of magnetic field^22,38,39,41^. Due to the limit configurations of the solenoid magnets, all these reported MR measurements were performed in a 2D plane without the third direction. To further reveal the anisotropic properties of MR, we carried out the 3D-space MR measurements. Figure 2(a) shows the schematic illustration of the 3D-space MR measurements. The current *I* is always applied along *a*-axis during the MR measurements. The magnetic field direction of 3D space is described by *α* (the angle between the magnetic field and *c*-plane) and *β* (the angle between the current and the *c*-plane projection direction of *B*). The MR is defined as [*ρ*(*B*)−*ρ*(0)]/*ρ*(0). Figure 2(b) shows the 3D-space MR mapping of 3 T at 1.7 K in ZrSiSe. From the MR mapping distribution, the AMR can be clearly seen. Corresponding color plot of the 3D-space MR is displayed in Fig. 2(c). At a certain *β*, the angular dependent MR almost exhibits the butterfly shape [Fig. 2(d)] with the continuous changing *α*, and the maximum values of MR appear near *α* = 45°, 135°, 225° and 315° directions. As *β* increases, MR at *α* = 45° family directions increases and finally reaches the maximum at *β* = 90°. To clearly see the angular dependent MR in *bc*-plane and *ac*-plane, Fig. 2(d) displays the polar plot of angular dependent MR of 3 T at *β* = 0° (black line) and *β* = 90° (red line). This reveals that the maximum MR occurs along [011] family directions, and the minimum MR appears along [100] family directions. The ratio of MR at maximum and minimum reaches 7 at 1.7 K and 3 T. The 3D-space MR mapping reveals the strong anisotropic electronic structures of ZrSiSe. The butterfly-shaped MR of *bc*-plane and *ac*-plane is the same as those observed in ZrSiS^22,38,39,41,48^. As reported in ZrSiS, the butterfly-shaped MR is regard as 2-fode and 4-fode symmetries^41^, which is very different from the AMR observed in WTe_2_^43,44^, LaBi^46^ and Bi^49,50^. For the perfect 2D Fermi surface structure, the angular dependent MR exhibits a typical 2-fold symmetry. In contrast, the MR is almost unchanged for the 3D isotropic electronic structure system^40^. Thus, the AMR in ZrSiSe is associated with the quasi-2D nature of the Fermi surface.

**Figure 3:**
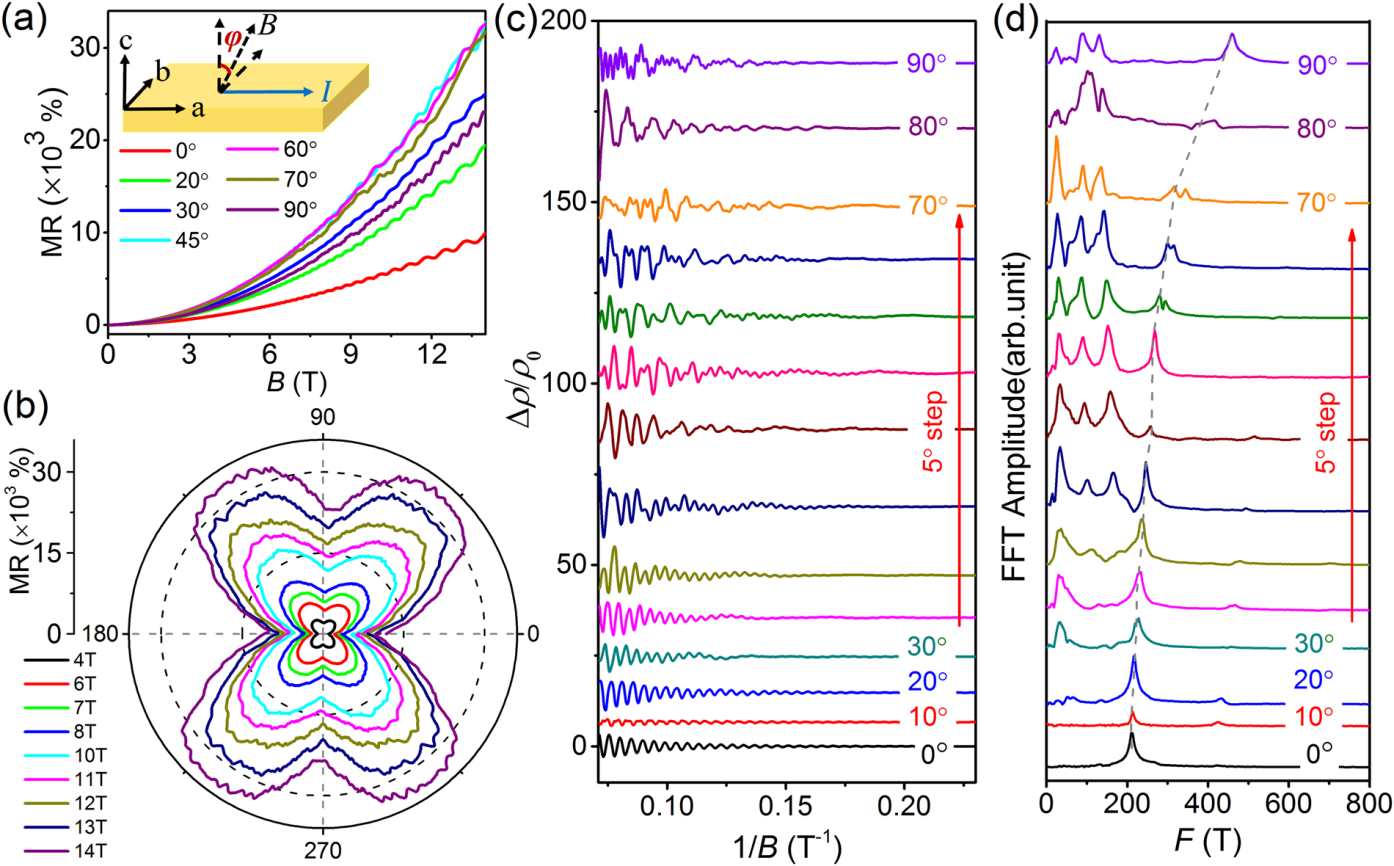
Angular dependent SdH oscillations of MR at 2.3 K with the magnetic field rotated in the bc-plane. (**a**) MR measured at the different angles with *φ* varying from 0° (*c*-axis) to 90° (*b*-axis). The inset shows the schematic diagram for the field rotation in the *bc*-plane. (**b**) Polar plot of angular dependent MR for the different magnetic fields along the *bc*-plane. (**c**) SdH oscillation amplitude (after polynomial background subtraction) measured with *φ* varying from 0°to 90°. (**d**) Corresponding FFT amplitude spectra of angular dependent SdH oscillations in the *bc*-plane.

To further reveal the origin of AMR in ZrSiSe, the angular dependent SdH oscillations can be used to obtain the shape of Fermi surface. We first measured MR curves at the different field directions along the *bc*-plane as the magnetic field is always perpendicular to the current direction to maintain a constant Lorentz force. The inset of Fig. 3(a) shows the schematic diagram for the *bc*-plane measurements. The magnetic field direction is indicated by *φ*, that is, the angle between the magnetic field and the *c*-axis on the *bc*-plane. The MR measurements with *φ* varying from 0° (*c*-axis) to 90° (*b*-axis) were carried out along the *bc*-plane at 2.3 K. As shown in Fig. 3(a), the SdH oscillations can be clearly seen at the different field direction. In order to clearly observe the difference of MR oscillations, we only depict MR curves corresponding to the different magnetic field directions. Figure 3(b) shows the polar plot of angular dependent MR from 4 T to 14 T at *bc*-plane, also exhibiting the clear butterfly-shaped anisotropy. When the magnetic field are increased to 7 T, there are some wiggles appearing at the maximum value of MR. The wiggles become more prominent as the field reaches 14 T, which is associated with the strong quantum oscillations of Landau level. As observed in WTe_2_^43^, the appearance of these additional oscillation wiggles is consistent with the observed SdH oscillations.

Figure 3(c) displays the amplitude of the angular dependent SdH oscillations with *φ* varying from 0° (*c*-axis) to 90° (*b*-axis) along the *bc*-plane at 2.3 K. The SdH oscillation amplitude Δ*ρ* [Fig. 3(c)] is obtained from MR measurements after the smooth polynomial background subtraction. For the better visibility, the oscillation amplitude is plotted in the form of Δ*ρ*/*ρ*_0_ (where *ρ*_0_ represents the resistivity at zero magnetic field), and the curves are shifted. Figure 3(d) shows the corresponding fast Fourier transformation (FFT) spectra of angular dependent SdH oscillations. We only observe a frequency oscillation of 210 T when the field lies along the *c*-axis (*φ* = 0°). It is evident that the *F* = 210 T frequency mode always exists and gradually shifts to 460 T with *ϕ* varies from 0° to 90°. In addition, the MR curves of other magnetic field directions with *ϕ* varies from 90° to 180° were also measured at *bc*-plane as shown in Fig. S5 (Supplemental Material). The MR and FFT spectrum with *ϕ* varying from 90° to 180° (Fig. S5) are almost the same as those measured results with *ϕ* varying from 0° to 90°.

**Figure 4:**
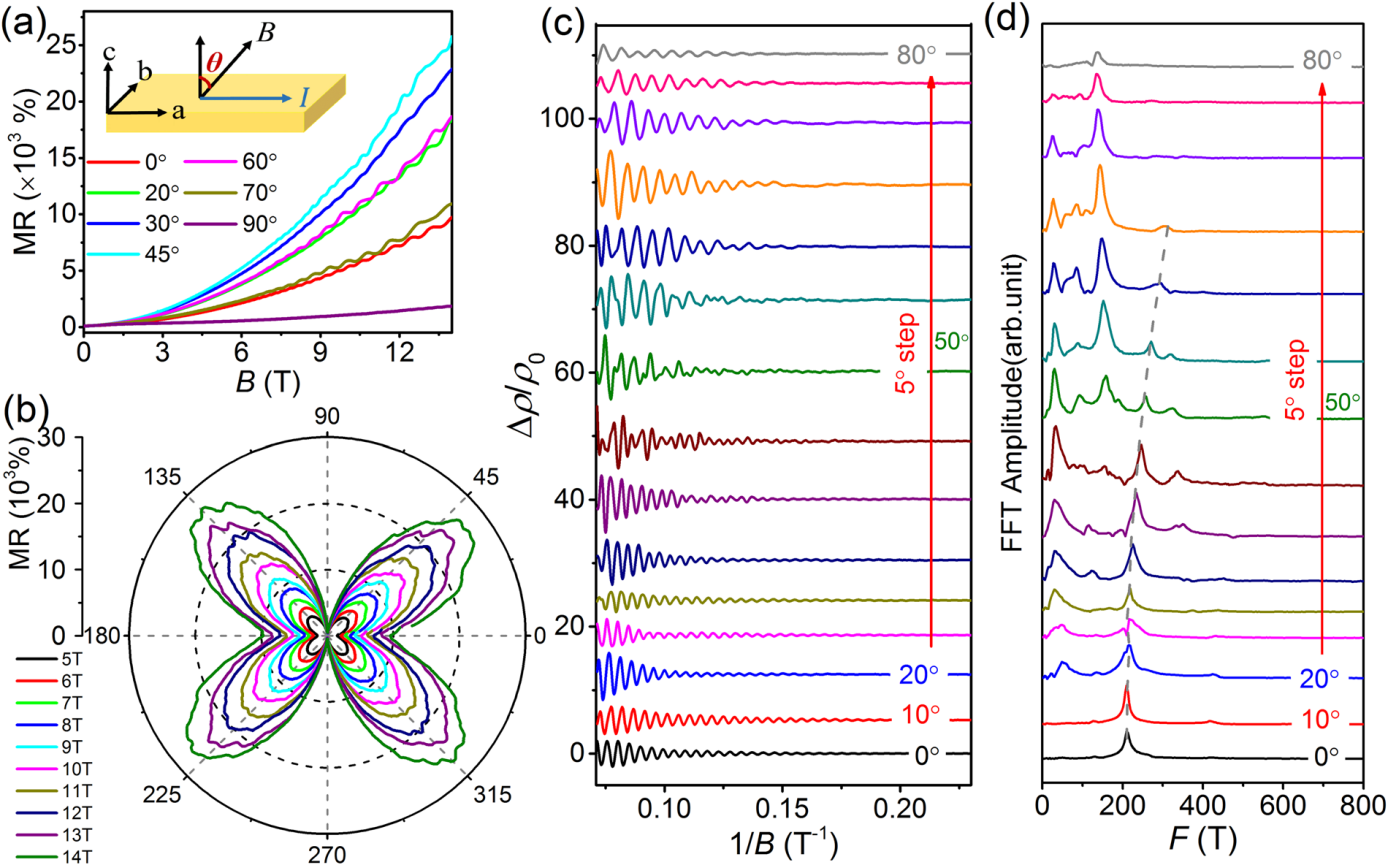
Angular dependent SdH oscillations of MR at 2.7 K with the magnetic field rotated in the ac-plane. (**a**) MR measured at the different angles with *θ* varying from 0° (*c*-axis) to 80° (near *a*-axis). The inset shows the schematic diagram for the field rotation in the *ac*-plane. (**b**) Polar plot of angular dependent MR for the different magnetic fields along the *ac*-plane. (**c**) SdH oscillation amplitude (after polynomial background subtraction) measured with *θ* varying from 0° to 80°. (**d**) Corresponding FFT amplitude spectra of angular dependent SdH oscillations in the *ac*-plane.

To construct the complete Fermi surface, we also performed the angular dependent MR measurements in the *ac*-plane at 2.7 K. The magnetic field is parallel to the current direction when *θ* = 90°. For clarity, only certain MR curves corresponding to the different angles are displayed in Fig. 4(a). Due to the effect of the non-uniform Lorentz force, the MR is very small, and SdH oscillations are unobservable as the magnetic field lies close to the *a*-axis. As shown in Fig. 4(b), the polar plot exhibits the strong anisotropy, similar to that of the *bc*-plane [Fig. 3(b)]. The Lorentz force has the little effect on the electron movement when the field lies along the *a*-axis, thereby resulting in an almost unchanged MR. Figure 4(c) shows the oscillation amplitude Δ*ρ*/*ρ*_0_ with *θ* varying from 0° to 80°. The different oscillation patterns are evident at various tilted angles, similar to the SdH oscillations in the *bc*-plane. The corresponding FFT spectra are displayed in Fig. 4(d). The shift of the *F* = 210 T frequency oscillation can be clearly seen. The MR curves and corresponding FFT spectrum with *θ* varying from 90° to 180° (Fig. S8) were also measured at *ac*-plane, exhibiting the same behavior as *θ* varying from 0° to 90°.

**Figure 5:**
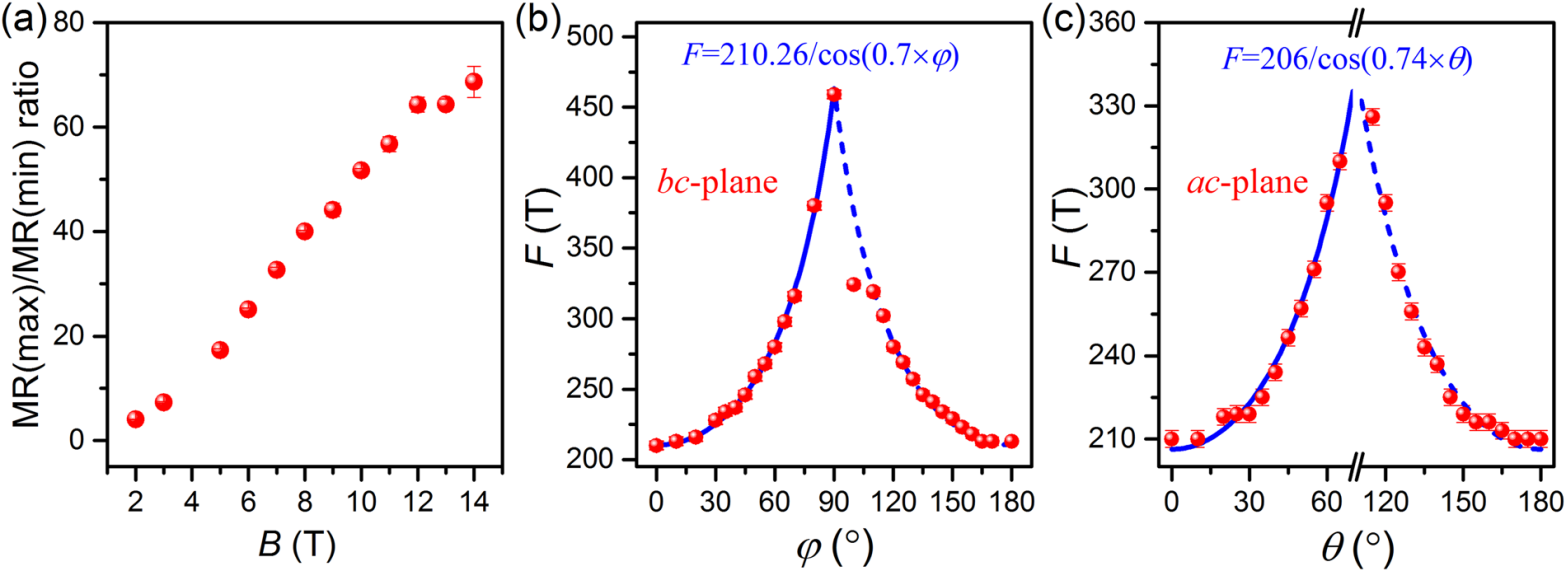
The anisotropic MR ratio and the evolution of SdH oscillation frequencies. (**a**) The ratio of MR at maximum and minimum versus the magnetic field. (**b**,**c**) Angular dependent SdH oscillation frequencies (red circles) in *bc*-plane and *ac*-plane, respectively. The blue solid and dashed lines are the frequencies fitted by *F* = *F*_1_/cos(*t* × *β*) and *F* = *F*_1_/cos[*t* × (180°− *β*)], respectively.

Taking the angular dependent MR in *bc*-plane and *a*c-plane together, we can estimate the MR ratio of maximum ([011] family directions) and minimum ([100] family directions). As shown in Fig. 5(a), the MR ratio keeps the linear increase with increasing magnetic field. There is even no saturation signal when the field increases to 14 T. The angular dependent frequency of 210 T SdH oscillations in *bc*-plane and *a*c-plane are plotted in Fig. 5(b) and Fig. 5(c), respectively. It is evident that the *F* = 210 T frequency mode always exists and gradually increases as *φ* and *θ* vary from 0° to 90°. For the 3D Fermi surface, the corresponding frequency is almost unchanged for the different tilted angles of the field^22,40,41^. For the 2D Fermi surface, the frequency and its corresponding cross-sectional areas follow the 1/cos(*β*) law at the different tilted angles of the field^22,40^. Hence, 3D and 2D characteristics coexist in the Fermi surface of ZrSiSe. The formula^41^ *F* = *F*_1_/cos(*t* × *β*) can be used to fit the *F* = 210 T frequency mode, where *F*_1_ and *t* denote the fitting parameters. *t* is determined by the dimensionality of the Fermi surface. *t* = 0 and 1 stand for the 3D and 2D cases, respectively. The tilted angle *β* equals *φ* or *θ*. The blue solid lines in Fig. 5(b) and Fig. 5(c) show the perfect fitting results, where the fitting parameters in *bc*-plane (*F*_1_ = 210.26 T and *t* = 0.70) and in *ac*-plane (*F*_1_ = 206 T, *t* = 0.74) are obtained. The dimensionality parameter (*t* = 0.70 and 0.74) locates between 0 and 1, indicating the coexistence of 2D and 3D characteristics for the *F* = 210 T frequency mode. In addition, a low oscillation frequency near *F* = 30 T is also observed with almost no shift in *bc*-plane and *a*c-plane, indicating the 3D nature of the Fermi surface. In addition to the high (210 T) and low (30 T) oscillation frequency modes, there also exist other frequency modes at the large-scale angles, which are related to the Fermi pockets near the Fermi level.

## Discussion

**Figure 6:**
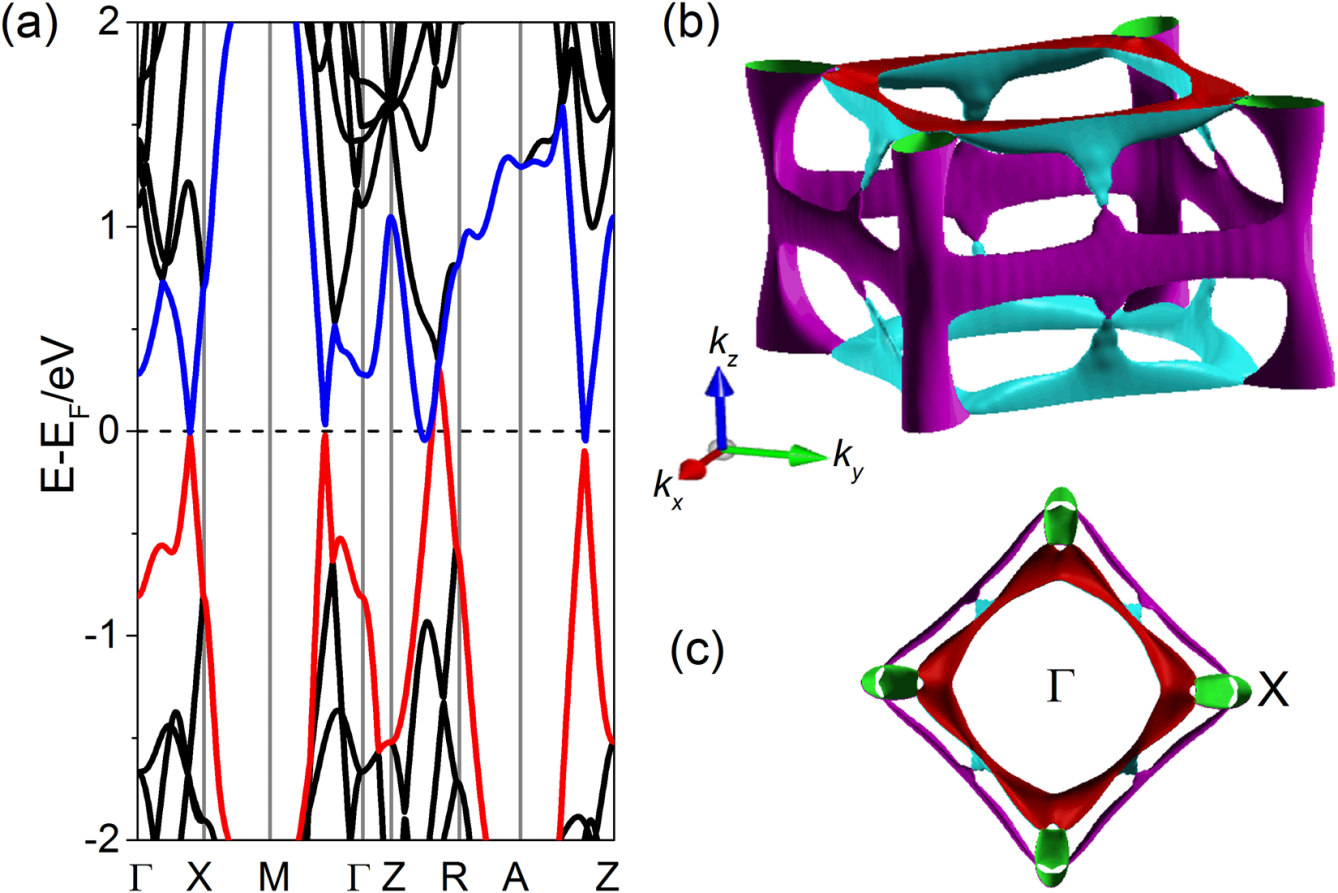
Calculated band structure and Fermi surface of ZrSiSe. (**a**) Calculated band structure along various high-symmetry directions. (**b**) Corresponding 3D Fermi surfaces of ZrSiSe in the reciprocal space. (**c**) Detailed Fermi surface observed along the *k*_*z*_ direction.

In order to fully discuss the origin of AMR, we performed the first-principles density functional theory (DFT) calculations to further study the properties of the electronic band structures and the Fermi surface of ZrSiSe. Figure 6(a) shows the calculated band structure of ZrSiSe along various high-symmetry directions in the BZ. The spin-orbit coupling is not considered in our DFT calculations^31^. Several Dirac cones are observed near the Fermi level, and Dirac-cone-like features are also visible below the Fermi level, similar to the calculated band structure of ZrSiS^34,51^. These band structure features indicate that electron and hole pockets coexist at the Fermi surface. The corresponding Fermi surface is displayed in Fig. 6(b). As shown in Fig. 6(c), the diamond-shaped Fermi surface formed by the linearly dispersing Dirac cone bands near the Fermi level emerges when along the *k*_*z*_ direction. The diamond-shaped Fermi surface is composed of four lens-shaped pockets. In addition, four small quasi-2D tubular-shaped Fermi pockets appear at the corners of the diamond-shaped Fermi surface (*X* locations in BZ). A similar quasi-2D electrical structure has also been reported in the tubular-shaped Fermi surface of ZrSiS^23,40,41^ and the cylindrical Fermi surface of CeCo_2_Ga_8_^52^. The diamond-shaped Fermi surface around *Γ* and small pockets near *X* point have been observed via ARPES measurements in ZrSiS and ZrSiSe^31,34^. The cross-sectional area of the observed small Fermi pocket near *X* point is ~3 × 10^−2^ Å^−2^ in ZrSiSe^31^, consistent with our calculated area order (10^−2^ Å^−2^). With the use of the Onsager relation, we obtain the cross-sectional area (*S*_*F*_ = 2 × 10^−2^ Å^−2^) of the Fermi surface associated with the *F* = 210 T frequency oscillation, in agreement with ARPES measurements at the *X* point in the BZ^31^. The cross-sectional area of lens-shaped pockets is evidently larger than that of the tubular-shaped Fermi pockets. This indicates that the *F* = 210 T frequency oscillation corresponds to the small quasi-2D tubular-shaped pocket at the *X* point. The high-field SdH-oscillation study in ZrSiS have revealed that the high frequency of 240 T arises from the petal-like Fermi surface pocket near the *X* point^51^. Ali *et al*. reported the high frequency of 243 T in ZrSiS originates from the tubular-shaped Fermi pocket near the *X* point^41^. Due to the usual band features of ZrSiSe, both the quasi-2D tubular-shaped Fermi pocket near the *X* point and other multiple band pockets at the Fermi level contribute to the unusual AMR transport behavior.

In conclusion, we have performed the 3D-space MR measurements in the DNLM ZrSiSe. The 3D-space MR mapping of 3 T and 1.8 K exhibits the strong anisotropy. The minimum and maximum of MR is observed when the magnetic field along [100] family directions and [011] family directions, respectively. The AMR in the main crystal planes keeps the butterfly-shaped curves with 2-fode and 4-fode symmetries up to 14 T. The ratio of MR at maximum and minimum is proportional to the magnetic field, indicating the stable AMR effect in ZrSiSe. Through angular dependent SdH oscillations and band structure calculations, the strong AMR behavior is associated with its anisotropic Fermi surface, providing the opportunity to achieve the magnetic sensor devices and explore the other potential technological applications.

## Methods

### Crystal Growth and Characterization

Single crystals of ZrSiSe were grown using the chemical vapor transport method with iodine (I_2_) as the transport agent^22^. Stoichiometric mixtures of Zr, Si, and Se powder were loaded into a quartz tube with I_2_ (5 mg/cm^3^). The quartz tube was evacuated, sealed, and placed in a horizontal tube furnace such that a temperature gradient was created from 950 °C to 850 °C. The end of the quartz tube was located in the high-temperature zone (950 °C), and the empty end of the quartz tube was maintained at 850 °C. The quartz tube was kept in the tube furnace for two weeks and then cooled down to room temperature. Finally, plate-like crystals with a metallic luster were obtained at the cold end. The structure of ZrSiSe single crystals was examined by means of a Bruker-D8 ADVANCE X-ray diffractometer using Cu-Kα radiation.

### Transport Measurements

Four-probe measurements of MR and Hall resistivity were carried out on the Quantum Design Physical Property Measurement System (PPMS-14). The 3D-space MR was measured on a vector magnet, on which the magnetic field value of vertical and horizontal directions can be changed simultaneously. The 3D-space magnetic field of a fixed value can be realized through rotating the sample and changing the field values of vertical and horizontal directions.

### Band Structure Calculations

Band structure of bulk ZrSiSe were calculated in the framework of DFT using the WIEN2K code with a full-potential linearized augmented plane-wave. The Perdew-Becke-Ernzerhof (PBE) parameterization of the Generalized Gradient Approximation (GGA) as the exchange-correlation functional was implemented. The irreducible BZ was sampled by a 15 × 15 × 7 mesh of *k*-points.

### Data availability

The data that support the findings of this study are available from the corresponding author upon reasonable request.

## Electronic supplementary material

Supplemental Materials
