# Supplementary material for: Three-Dimensional Anisotropic Magnetoresistance in the Dirac Node-Line Material ZrSiSe
Source: Sci Rep. 2018 Jun 19;8:9340. doi: 10.1038/s41598-018-27148-z (PMC6008472; doi:10.1038/s41598-018-27148-z)
Supplement: Supplementary file 1 — Supplemental Materials [file 41598_2018_27148_MOESM1_ESM.docx]

**Supplemental Material**

**Three-Dimensional Anisotropic Magnetoresistance in the Dirac Node-Line Material ZrSiSe**

Haiyang Pan^1^, Bingbing Tong^2^, Jihai Yu^1^, Jue Wang^1^, Dongzhi Fu^1^, Shuai Zhang^1^, Bin Wu^1^, Xiangang Wan^1,4^, Chi Zhang^2^, Xuefeng Wang^3,4^ & Fengqi Song^1,4^

^1^National Laboratory of Solid State Microstructures, School of Physics, Nanjing University, Nanjing 210093, China

^2^International Center for Quantum Materials, Collaborative Innovation Center of Quantum Matter, Peking University, Beijing 100871, China

^3^School of Electronic Science and Engineering, Nanjing University, Nanjing 210093, China

^4^Collaborative Innovation Center of Advanced Microstructures, Nanjing University, Nanjing 210093, China


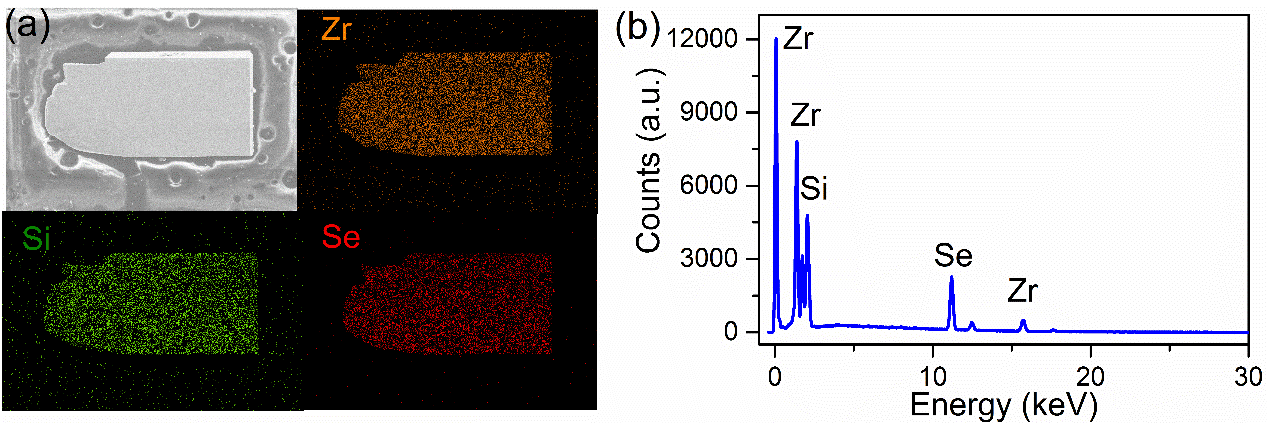


**Fig. S1**. energy-dispersive X-ray spectroscopy (EDS) analysis of a typical ZrSiSe bulk crystal. **a** scanning electron microscopy (SEM) image and the Zr, Si, and Se elemental mapping images. **b** The EDS spectrum shows the atomic ratio of Zr:Si:Se about 1:1:1.


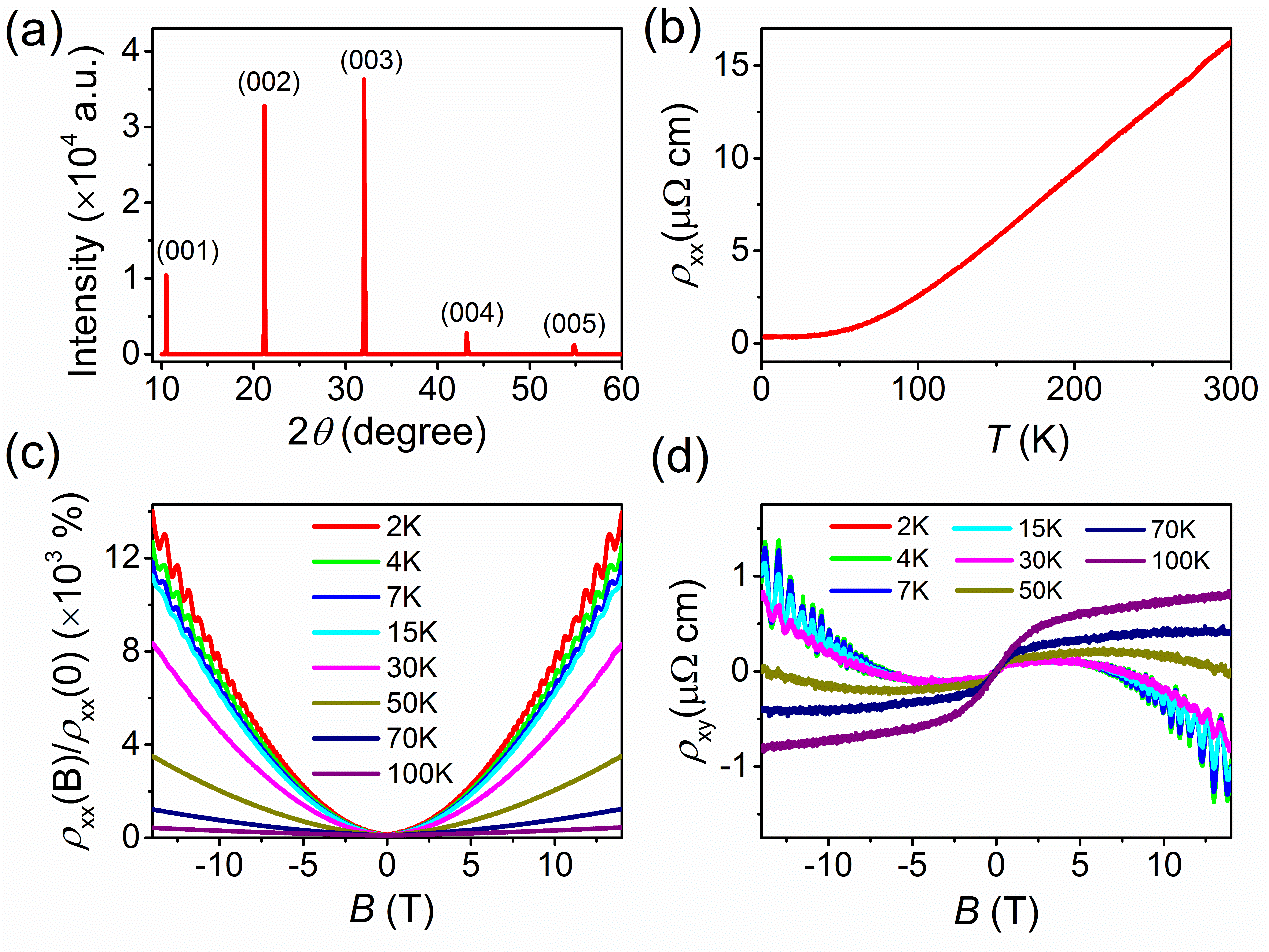


**Fig. S2**. X-ray diffraction and electrical transport in ZrSiSe bulk crystals. **a** X-ray diffraction (XRD) patterns of a typical single crystal of ZrSiSe. The XRD peak position indicates that the sample surface lies along the (001) plane. **b** Temperature-dependent longitudinal resistivity at zero magnetic field. **c** Ratio of MR *ρ*_xx_(B)/*ρ*_xx_(0) at different temperatures. The magnetic field is applied along (001) direction. **d** Corresponding Hall resistivity measured at different temperatures. The nonlinear-Hall-signal-dependent magnetic field reveals multiband features.


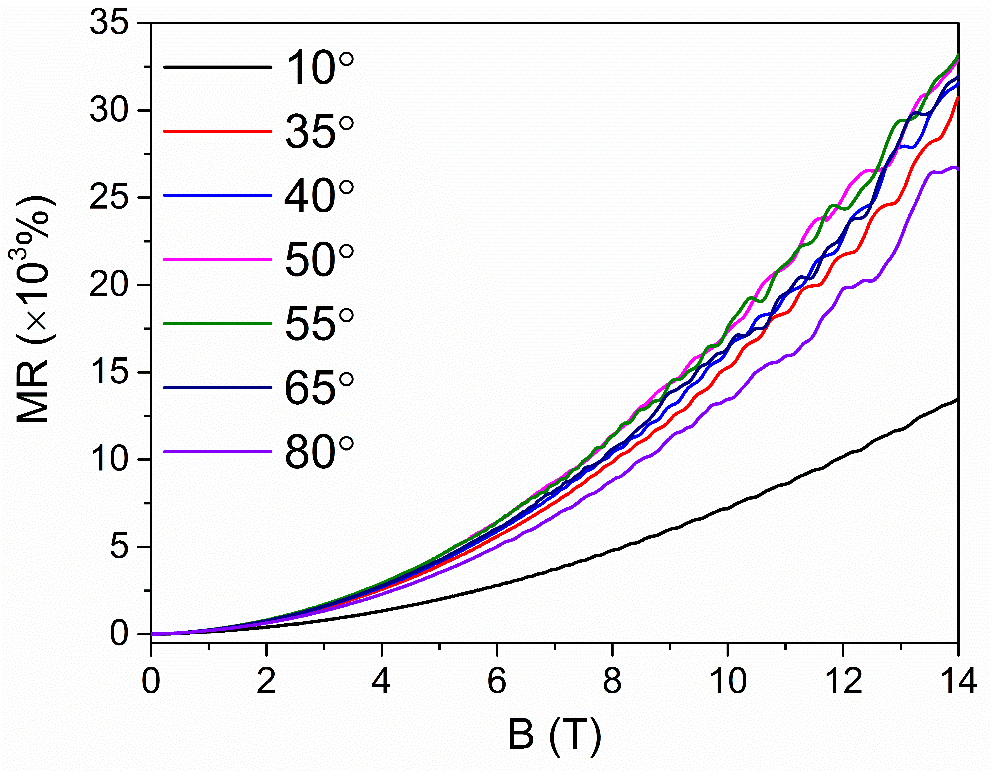


**Fig. S3**. The rest MR curves in *bc*-plane with *φ* varying from 0° (*c*-axis) to 90° (*b*-axis).


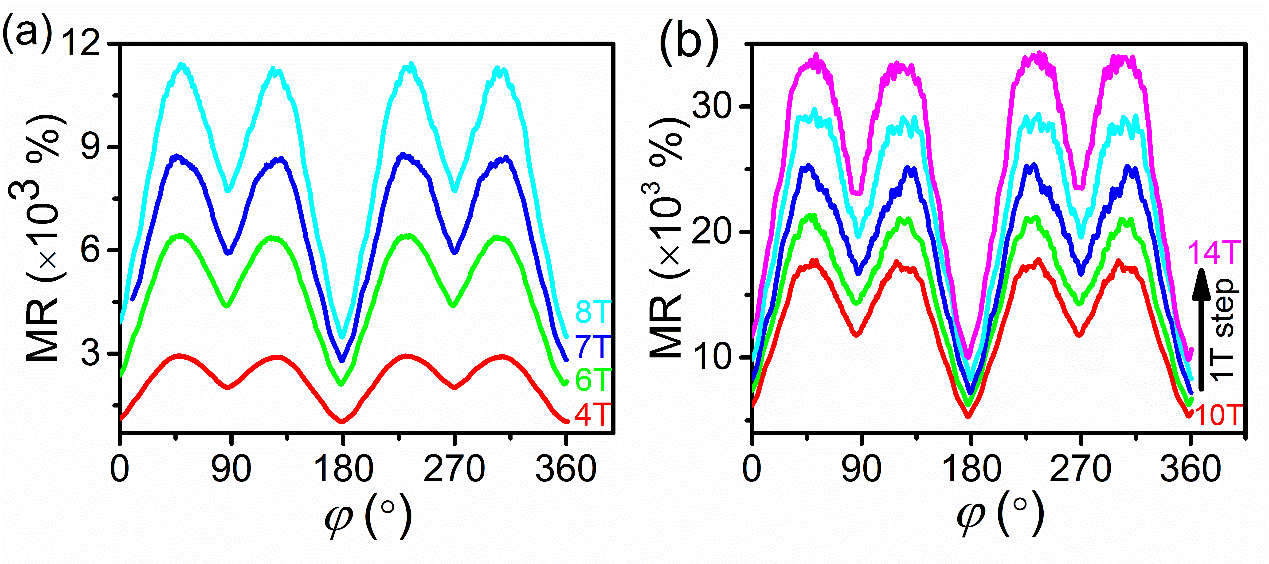


**Fig. S4**. The anisotropy MR of higher magnetic field with *B* rotated in the *bc*-plane at 2.3 K. **a** below 10 T. **b** above 10 T.


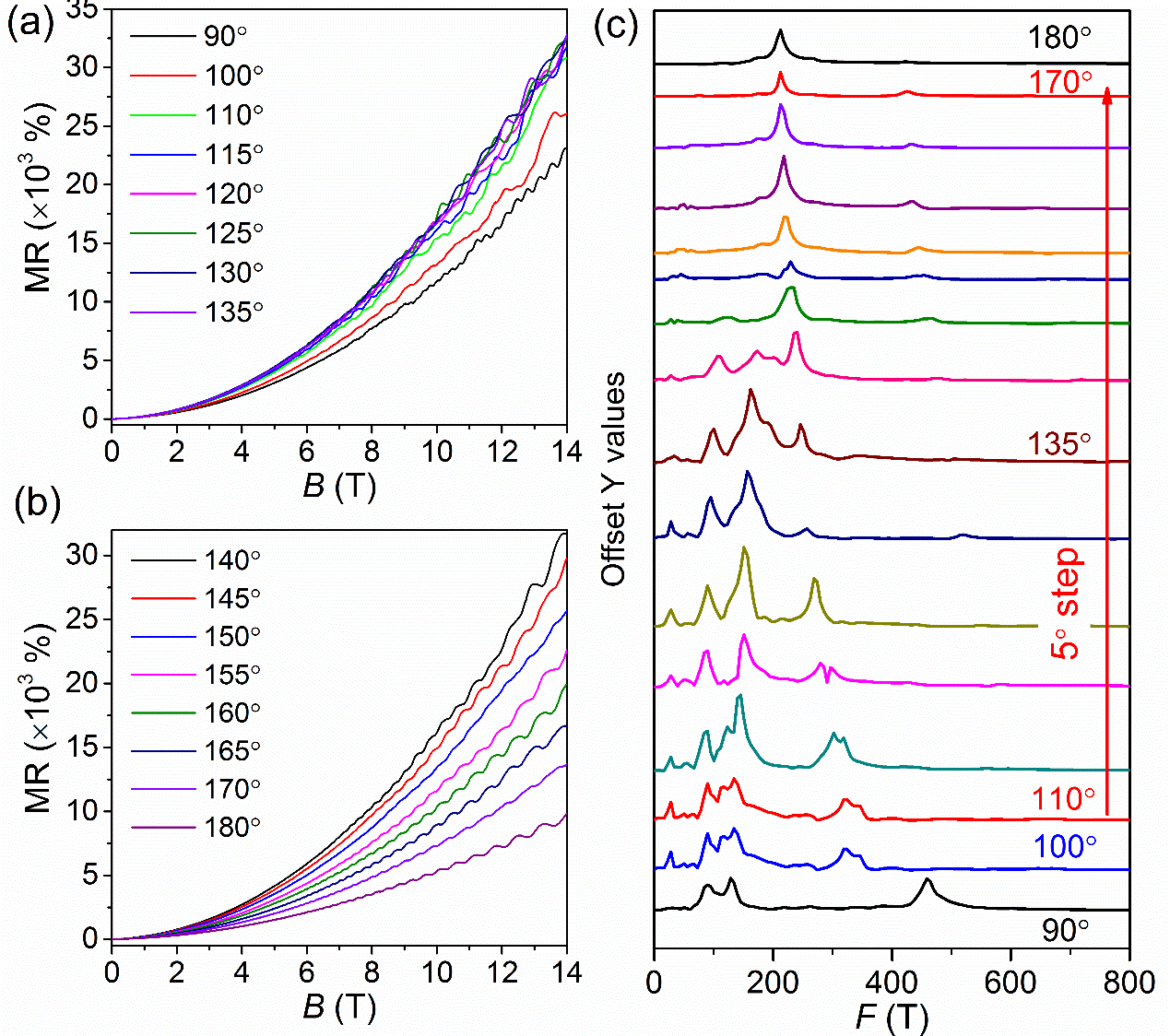


**Fig. S5**. Angular-dependent Shubnikov–de Haas (SdH) oscillations of transverse magnetoresistance (MR) at 2.3 K with magnetic field *B* rotated in the *bc*-plane. **a** and **b** MR measured at different angles with *φ* varying from 90° (*c*-axis) to 180° (*b*-axis). **c** Corresponding fast Fourier transform (FFT) amplitude spectra of angular-dependent SdH oscillations in the *bc*-plane.


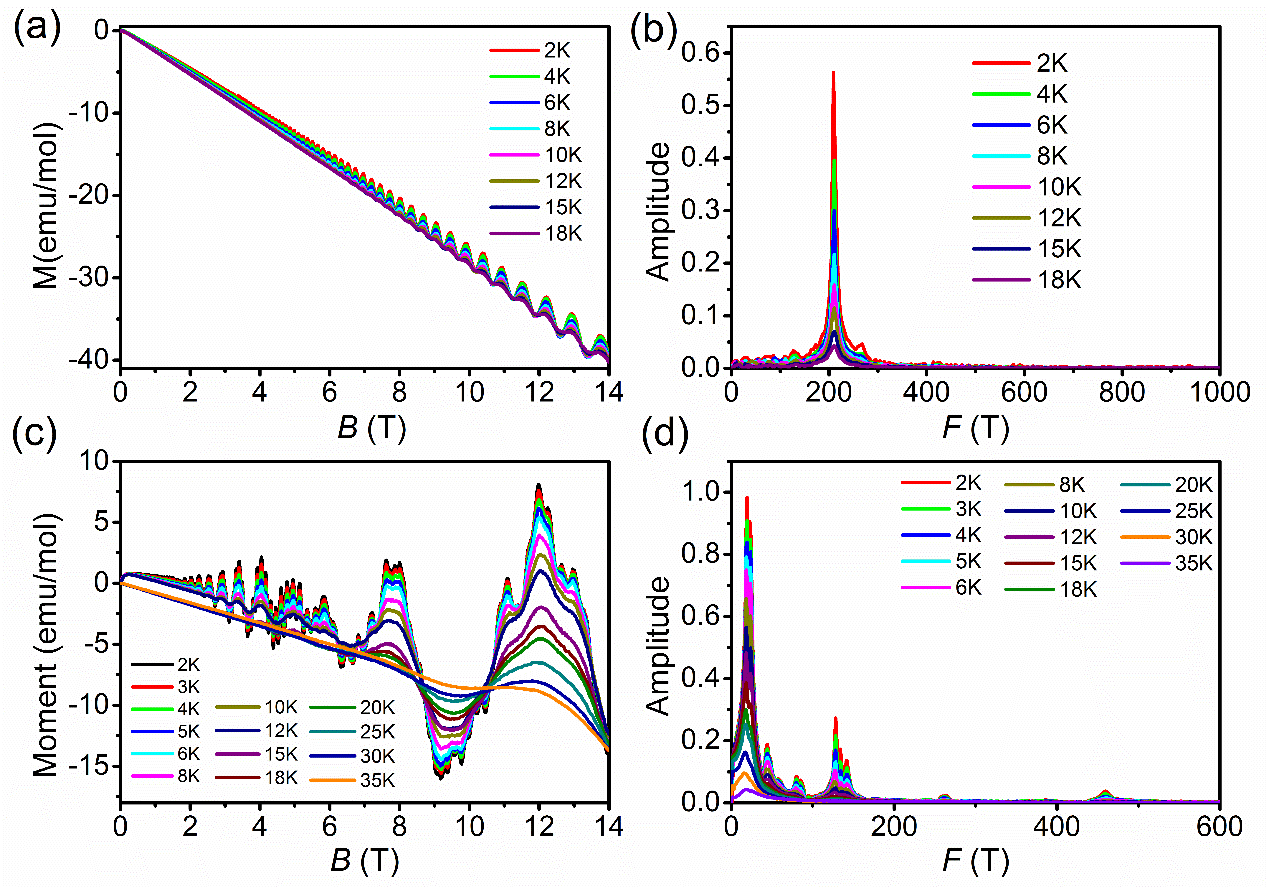


**Fig. S6**. de-Haas-van Alphen quantum oscillations of ZrSiSe crystals. **a** Isothermal out-of-plane (*B*//*c*-axis) magnetization measured at different temperatures. **b** Corresponding FFT of out-of-plane at different temperatures. **c** Isothermal in-plane (*B*//*ab*-plane) magnetization measured at different temperatures. **d** Corresponding FFT of in-plane at different temperatures.


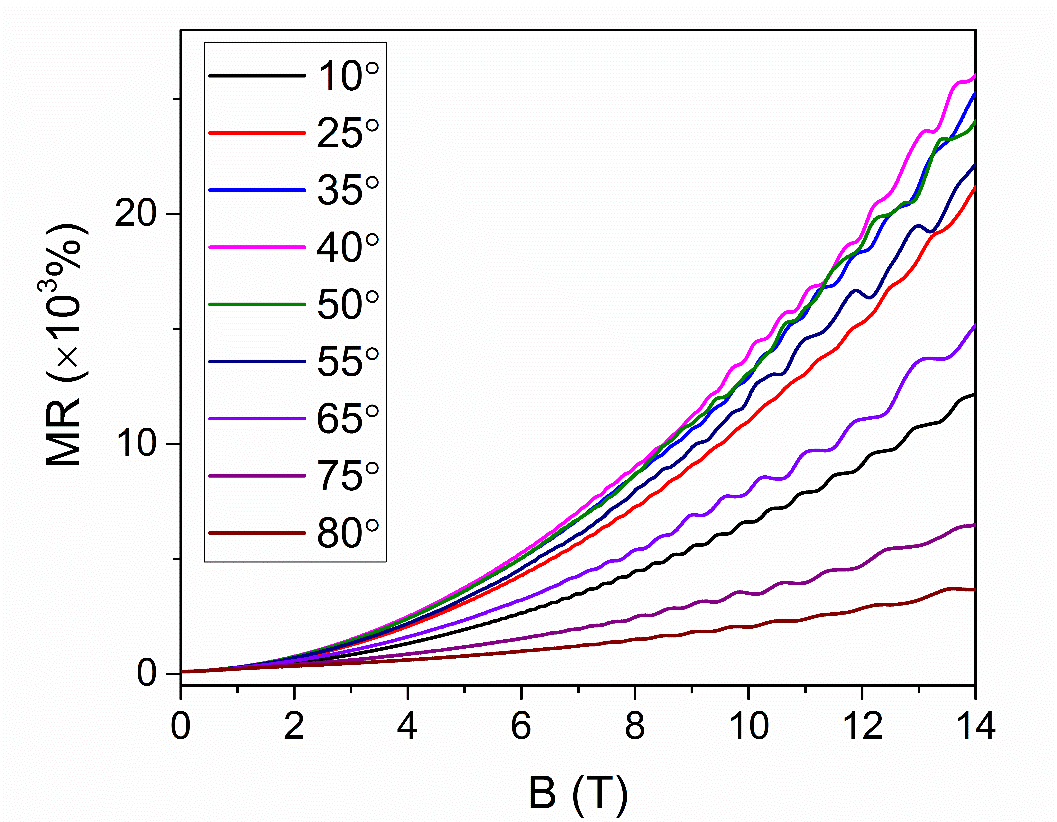


**Fig. S7**. The rest MR curves in *ac*-plane with *θ* varying from 0° (*c*-axis) to 90° (*a*-axis).


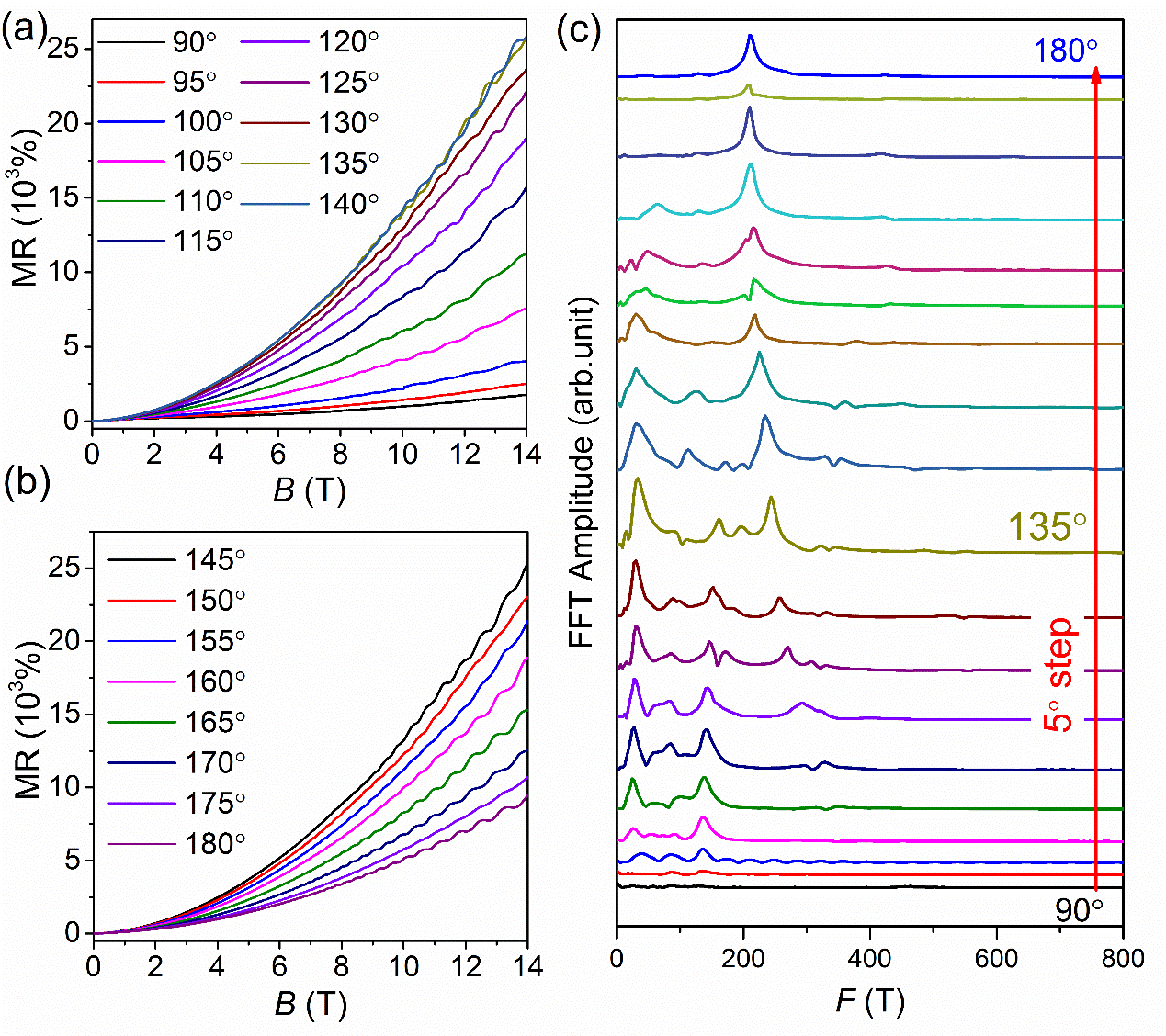


**Fig. S8**. Angular-dependent Shubnikov–de Haas (SdH) oscillations of transverse magnetoresistance (MR) at 2.3 K with magnetic field *B* rotated in the *ac*-plane. **a** and **b** MR measured at different angles with *θ* varying from 90° (*c*-axis) to 180° (*a*-axis). **c** Corresponding fast Fourier transform (FFT) amplitude spectra of angular-dependent SdH oscillations in the *ac*-plane.


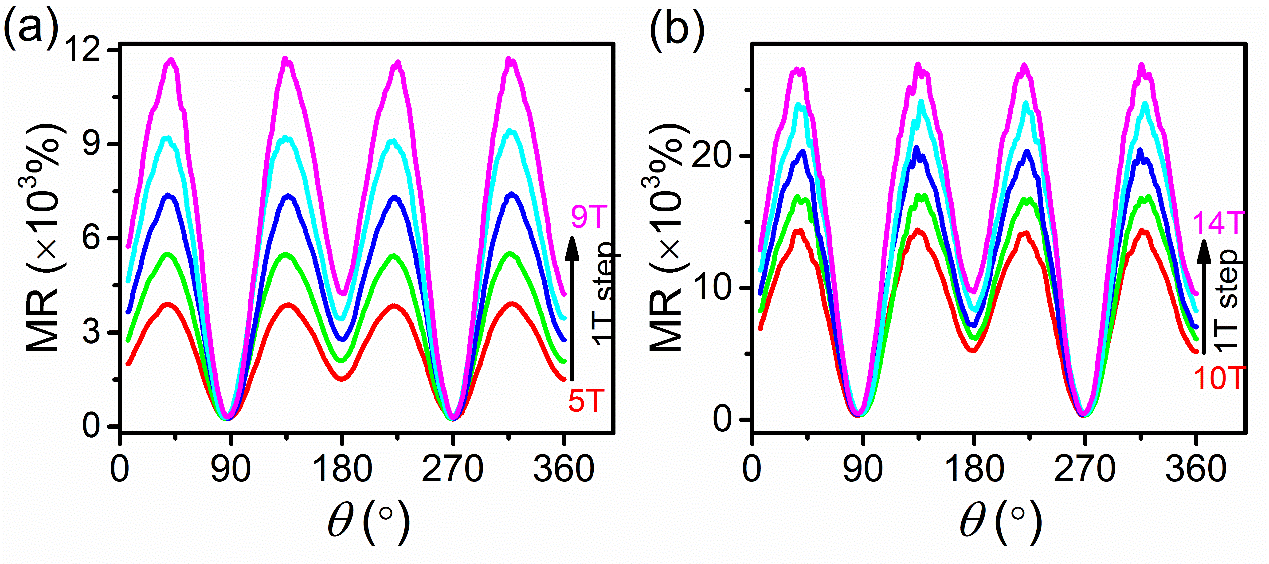


**Fig. S9**. The anisotropy magnetoresistance (MR) of higher magnetic field with *B* rotated in the *ac*-plane at 2.7 K. **a** The angular-dependent MR replotted of Figure 4b below 10 T magnetic field values. **b** The angular-dependent MR replotted of Figure 4b above 10 T magnetic field values.
